# The Na/K-ATPase Signaling: From Specific Ligands to General Reactive Oxygen Species

**DOI:** 10.3390/ijms19092600

**Published:** 2018-09-01

**Authors:** Rebecca D. Pratt, Cameron R. Brickman, Cameron L. Cottrill, Joseph I. Shapiro, Jiang Liu

**Affiliations:** Departments of Biomedical Sciences and Medicine Joan C. Edwards School of Medicine, Marshall University, Huntington, WV 25755, USA; martin570@live.marshall.edu (R.D.P.); brickman@live.marshall.edu (C.R.B.); cottrill41@live.marshall.edu (C.L.C.); shapiroj@marshall.edu (J.I.S.)

**Keywords:** Na/K-ATPase, ROS, sodium, potassium, signaling, Src. endocytosis

## Abstract

The signaling function of the Na/K-ATPase has been established for 20 years and is widely accepted in the field, with many excellent reports and reviews not cited here. Even though there is debate about the underlying mechanism, the signaling function is unquestioned. This short review looks back at the evolution of Na/K-ATPase signaling, from stimulation by cardiotonic steroids (also known as digitalis-like substances) as specific ligands to stimulation by reactive oxygen species (ROS) in general. The interplay of cardiotonic steroids and ROS in Na/K-ATPase signaling forms a positive-feedback oxidant amplification loop that has been implicated in some pathophysiological conditions.

## 1. Introduction

Since J.C. Skou’s discovery in 1957 [1], the energy-transducing Na/K-ATPase has been extensively studied for its ion-pumping function and, later on, its signaling function. The latter was first demonstrated about two decades ago and evolved into a much bigger signaling network (and has kept evolving) that one could not imagine before.

All starts with the possible role of Na/K-ATPase in cardiac hypertrophy. In a classic view, partial inhibition of Na/K-ATPase ion-exchange activity raises intracellular sodium concentration ([Na^+^]_i_), which in turn increases intracellular calcium concentration ([Ca^2+^]_i_) by coupling with Na^+^/Ca^2+^ exchanger (NCX) to execute the inotropic effect. This is the basis of the treatment of heart failure with digitalis-like drugs. Furthermore, partial inhibition of Na/K-ATPase not only causes intracellular ionic changes but also stimulates transcriptional upregulation of several marker genes including Na/K-ATPase itself. However, further studies were unable to directly link ouabain-mediated gene regulation effects to changes in intracellular [Na^+^]_i_ or [K^+^]_i_ caused by Na/K-ATPase inhibition. In cultured cardiac myocytes, treatment with nontoxic concentrations of ouabain not only partially inhibited Na/K-ATPase activity and increased cardiac contractility but also stimulated cell growth and protein synthesis through induction of early response proto-oncogenes and activation of transcription factors [2,3,4,5,6,7,8]. These discrepancies started the search for mechanism(s) other than ionic changes.

## 2. Na/K-ATPase Signaling and Intracellular Ionic Concentration

As mentioned above, changes in [Na^+^]_i_, [K^+^]_i_, and [Ca^2+^]_i_ were largely attributed to changes in Na/K-ATPase activity that can be regulated by specific-ligand cardiotonic steroids. Interestingly, Na/K-ATPase activity can also be regulated by changes in cellular redox status, induced by either cardiotonic steroids or other factors. In cardiac myocytes, inhibition of Na/K-ATPase ion-exchange function leads to a decrease of [K^+^]_i_ and an increase of [Na^+^]_i_. By coupling to NCX, this increase in [Na^+^]_i_ elevates intracellular [Ca^2+^]_i_, which is the leading force of the positive inotropic action induced by digitalis drugs for treatment of heart failure [9,10]. To address the role of these ionic changes, ouabain-induced reactive oxygen species (ROS) generation (an essential second messenger) and an increase in [Ca^2+^]_i_ (a shared secondary messenger) were manipulated to investigate the possible interplay. Ouabain-induced ROS generation was compared in cardiac myocytes cultured in Ca^2+^-free medium (with 0.1 mM egtazic acid (EGTA)) and Ca^2+^-containing medium, respectively. In neonatal cardiac myocytes cultured in Ca^2+^-free medium, in which ouabain did not change [Ca^2+^]_i_, ouabain was still able to stimulate ROS generation as shown in myocytes cultured in Ca^2+^-containing medium but was unable to stimulate an increase of [Ca^2+^]_i_ and contractility in neonatal cardiac myocytes [11]. Furthermore, in neonatal cardiac myocytes cultured in Ca^2+^-free medium with high Na^+^ (150 mM), monensin, a Na^+^-specific ionophore capable of equilibrating Na^+^ concentration across cell membrane, failed to increase ROS generation. Interestingly, inhibition of c-Src or Ras as well as antioxidants can block ouabain-stimulated ROS generation but not ouabain-induced increases in [Ca^2+^]_i_ [6,12,13]. These observations suggest that increases in [Ca^2+^]_i_ are necessary in ouabain-induced increases in cardiomyocytes contractility and gene regulatory effects but is not necessary in ouabain-stimulated ROS generation. Moreover, ouabain-stimulated Na/K-ATPase signaling also increases the generation of ROS, which functions as a second messenger. Pretreatment with antioxidants, such as *N*-acetylcysteine (NAC) or vitamin E, neutralized the increases of ROS and therefore prevented ouabain-stimulated activation of NF-κB and protein synthesis [6,11]. Ouabain-induced increases in ROS production involves the opening of mitochondrial ATP-sensitive K^+^ channels (mitoK_ATP_) [11,13].

Notably, ouabain-induced increases in [Ca^2+^]_i_ are also involved in ouabain-stimulated Na/K-ATPase signaling. In renal epithelial cells, low doses of ouabain, which only partially inhibit Na/K-ATPase activity, functioned as an inducer/trigger of regular, low-frequency [Ca^2+^]_i_ oscillations, which are involved in the Na/K-ATPase/inositol 1,4,5-trisphosphate receptors (IP_3_Rs) signaling microdomain that leads to NF-κB activation [14,15]. This phenomenon does not depend on partial inhibition of Na/K-ATPase using low extracellular K^+^ and depolarization of cells but is achieved by ouabain-stimulated activation of tyrosine kinase c-Src and phospholipase C-γ (PLC-γ), which transmit the signal to IP3Rs [16,17]. Depletion of intracellular endoplasmic reticulum (ER) Ca^2+^ by sarco/endoplasmic reticulum Ca^2+^ ATPase (SERCA) inhibitor, as well as blockage of store-operated calcium-mediated cytosolic Ca^2+^ influx and inhibition of IP3Rs-induced Ca^2+^, release abolished ouabain-induced Ca^2+^ oscillations [15]. Truncation of 32 amino acids from the α1 NH_2_ terminus results in a functional enzyme but abolishes ouabain-induced Ca^2+^ oscillations, indicating that the cytoplasmic α1 NH_2_ terminus plays a central role in ouabain-induced Ca^2+^ oscillations [15]. The data from this study also indicates that increased [Na^+^]_i_ is not the main cause of ouabain-induced Ca^2+^ oscillations, but rather the release of the α1 NH_2_ terminus during the Na/K-ATPase E1 to E2 conformational change (ouabain binding favors E2 conformation) serves as a mean of α1/IP3Rs complex formation. It was further confirmed that the α1 NH_2_ terminus binds directly, through motif LKK, with the IP3R NH_2_ terminus [18]. On the other hand, ouabain stimulated the formation of a functional Ca^2+^-signaling complex, including the Na/K-ATPase α1/c-Src/PLC-γ/IP_3_R in LLC-PK1 cells, and knockdown of the Na/K-ATPase α1 redistributed IP3R [17]. Overexpression of the α1 NH_2_ terminus (amino acids 1–160) not only disrupted the interaction of the Na/K-ATPase α1 and IP3R, but it also functioned as a negative regulator of ATP-induced ER Ca^2+^ release [17]. The p42/44 MAPK-mediated activation of Ca^2+^ channels partially contributes to ouabain-induced regulation of [Ca^2+^]_i_ [19,20].

Because the effects of ouabain on c-Src are independent of changes in intracellular ion concentrations [11,21,22,23], it seems that ouabain-induced inhibition of Na/K-ATPase enzymatic activity (ion exchange) and ouabain-induced c-Src-dependent signaling are, at least partially, two separated regulatory events under these experimental conditions. In addition to ouabain, changes in intracellular Na^+^ or extracellular K^+^ affect not only the enzyme conformation but also change other ion-transporter-related activities. For example, lowering of extracellular K^+^ activates protein kinases and raises intracellular Ca^2+^ in cardiac myocytes [23], but it differs from the effects of ouabain on smooth muscle and kidney epithelial cells [14,21]. Large bulk changes in [Na^+^]_i_ or [K^+^]_i_ that are not induced by the ouabain–Na/K-ATPase axis also affect other intracellular signal pathways [24,25,26]. For example, in cultured porcine aortic endothelial cells, ouabain-mediated complete inhibition of Na/K-ATPase causes cell necrosis that is independent of ouabain-mediated ion fluxes and changes of the [Na^+^]_i_/[K^+^]_i_ ratio, but K^+^-free ([K^+^]_0_ = 0) medium-caused inhibition of Na/K-ATPase, which elevates [Na^+^]_i_, does not induce necrosis but protects against apoptosis [27]. The antiapoptosis effect is regulated by a [Na^+^]_i_-mediated, Ca^2+^-independent mechanism [28]. In cultured cortical neurons, ouabain-induced concentration-dependent neuron death involved apoptosis and necrosis, which is mediated by intracellular depletion of K^+^ and accumulation of Ca^2+^ and Na^+^ [29]. These observations suggest that ouabain-Na/K-ATPase-mediated signaling differs from solely ionic change **(**[Na^+^]_i_/[K^+^]_i_**)-**mediated signaling pathways. The possible interplay and different mechanism(s) between them are still not totally understood.

In smooth muscle cells of rat arteries, both NCX and ouabain-sensitive Na^+^/K^+^-ATPase α_2_- and α_3_-isoforms reside closely in plasma membrane regions adjacent to the sarcoplasmic reticulum, a subplasmalemmal space called plasmERosome [30,31,32,33]. While low doses of ouabain do not increase bulk cytosolic Na^+^ levels, it can stimulate a local transient rise of [Na^+^]_i_ in the plasmERosome, which can lead to a local transient increase of [Ca^2+^]_i_ via the NCX and increased muscle contractility [34,35,36].

In cells without expression of plasma membrane NCX, alteration of the [Na^+^]_i_/[K^+^]_i_ ratio, by high ouabain concentrations or palytoxin through inhibition of Na^+^/K^+^-ATPase, is able to activate some protein kinase signaling pathways [25,26,27,28,29,30,31,32,33,34,35,36,37]. This suggests that, in cells lacking NCX expression, changes in [Na^+^]_i_ or [K^+^]_i_ or both may also stimulate Ca^2+^-independent Na^+^/K^+^-ATPase signaling functions.

These observations indicate a complicated interplay amongst Na/K-ATPase ion-exchange activity, signaling, and ROS in regulation of different cellular events. The Na/K-ATPase signaling–ROS axis might play an important role in dissecting these regulations since chronic regulation of ion homeostasis could be a consequence of Na/K-ATPase signaling and ROS regulation.

## 3. Na/K-ATPase Signaling and Reactive Oxygen Species (ROS): The Positive Oxidant Amplification Loop

The interplay amongst Na/K-ATPase signaling, ROS, and oxidative modifications has been a topic for decades. The effect of ROS on Na/K-ATPase activity has been well documented [6,38,39,40,41,42,43,44,45,46]. Different oxidative modification mechanisms and subunits of the Na/K-ATPase showed different outputs.

In rabbit ventricular myocytes, glutathionylation of cysteine residue (Cys-46) of the Na/K-ATPase β1 subunit inhibits Na/K-ATPase activity by either stabilizing the enzyme in an E2-prone conformation, a process that could be reversibly regulated by glutaredoxin 1 and FXYD proteins (a family of seven type I small membrane proteins sharing a 35 amino acid signature domain starting with PFXYD) that are associated with Na/K-ATPase [40,41,47]. In rat myocardium, *S*-glutathionylation of cysteine residues (Cys-454, -458, -459, and-244) of the Na/K-ATPase α1 subunit also inhibits the Na/K-ATPase activity by blocking the ATP-binding site Na/K-ATPase α1 subunit when the ATP concentration below 0.5 mM. This *S*-glutathionylation of the α1 subunit as well as inhibition of Na/K-ATPase activity can be reversed by deglutathionylation with glutaredoxin or dithiothreitol [42,48]. In various chronic inflammatory conditions, circulating cardiotonic steroids are elevated that are capable of stimulating a proinflammatory response in murine and human macrophages. This process involves ouabain-stimulated activation of NF-κB through a signaling complex of Na/K-ATPase, CD36, and TLR4, leading to increases in proinflammatory cytokines MCP-1, TNF-α, IL-1β, and IL-6 [49,50,51].

In rat neonatal myocytes, ouabain-stimulated activation of the Na/K-ATPase signaling function increases mitochondrial ROS generation that functions as an essential second messenger [6,11]. More importantly, increases in ROS can cause conformational changes in Na/K-ATPase like ouabain [39,40,41,42,44,47]. One question asked was that if ROS is able to stimulate the signaling function of Na/K-ATPase like ouabain, and if ouabain (or ROS) → Na/K-ATPase signaling → ROS → Na/K-ATPase signaling could form a positive amplification loop that could amplify Na/K-ATPase and subsequent signaling events and functional changes. This is of particular interest since it was well accepted that ROS play an important role in the pathogenesis of cardiovascular diseases, chronic kidney diseases, and many others.

In primary cultures of cardiac myocytes, it was demonstrated that partial inhibition of Na/K-ATPase by ouabain stimulated c-Src- and Ras-dependent signaling which lead to mitochondrial K_ATP_ channel-related ROS generation, and that ouabain-induced cardiac hypertrophic growth involved ROS-dependent signaling pathways [6]. In Langendorff-perfused rat hearts, pretreatment with ouabain demonstrated a cardioprotective effects against ischemia-reperfusion injury by an improved recovery of contractile function and a reduction of infarct size. This ouabain effect is due to activation of the Na/K-ATPase signaling function that involves Src, the mitochondrial K_ATP_ channel, and ROS [52]. Exogenous ROS (for example, induced by glucose oxidase) acting as ouabain also caused ROS-dependent cardiac hypertrophic growth. Inhibition of c-Src and ERK1/2 abrogated the effects of ROS-induced protein synthesis that was not affected by chelating intracellular Ca^2+^ by BAPTA-AM [53]. Moreover, ouabain-induced increase in [Ca^2+^]_i_ was ROS-independent and involved mainly the inhibition of the Na/K-ATPase ion transport function [11]. These observations indicated that ROS act like ouabain, and the Na/K-ATPase could be a target for ROS-initiated signaling.

In porcine LLC-PK1 cells (an immobilized renal proximal tubule cell line), exogenous H2O2 activated Na/K-ATPase signaling pathways including phosphorylation of c-Src and ERK1/2 [54]. By using LLC-PK1 cells, it was further demonstrated that a low concentration of ouabain also stimulated the Na/K-ATPase signaling function, which led to increased ROS generation and protein carbonylation modification of Na/K-ATPase (direct carbonylation of two amino acid residues, Pro222 and Thr224, in the actuator domain of the α1 subunit) [45]. Pretreatment with antioxidant *N*-acetyl-l-cysteine (NAC) or disruption of the Na/K-ATPase/c-Src signaling complex attenuated ouabain- and glucose-oxidase-stimulated Na/K-ATPase/c-Src signaling, protein carbonylation, redistribution of Na/K-ATPase, and inhibition of active transepithelial ^22^Na^+^ transport. This indicated that ROS are critical in initiating ouabain-stimulated Na/K-ATPase/c-Src signaling, and carbonylation modification of the α1 subunit is involved in a feed-forward mechanism of regulation of ouabain-mediated Na/K-ATPase signal function and subsequent Na^+^ transport. Interestingly, there is an undefined “decarbonylation” mechanism of ouabain-stimulated protein carbonylation after removal of ouabain, which could be another new regulatory mechanism of Na/K-ATPase signaling because it was believed that protein carbonylation modification could not be reversed. Furthermore, stable overexpression of rat α1 mutant Pro224/Ala (Pro224 of rat α1 is the same as the Pro222 of pig α1) prevented the ouabain-stimulated signal function of Na/K-ATPase, protein carbonylation, Na/K-ATPase endocytosis, and active transepithelial ^22^Na^+^ transport [46]. Taken together, we proposed that, in LLC-PK1 cells, there is a positive-feedback amplification loop of Na/K-ATPase signaling and ROS generation, in which carbonylation of the Pro222 of the α1 subunit plays a critical role. In this working model, both Na/K-ATPase specific-ligand cardiotonic steroids (including ouabain) and ROS increases (induced by other stimuli, including exogenous-added glucose oxidase) could activate Na/K-ATPase signaling. The Na/K-ATPase/c-Src complex functions as a “receptor” of ROS signaling. This Na/K-ATPase signaling–ROS axis may explain the role of Na/K-ATPase signaling in the development of different pathophysiological conditions. However, it is not clear (1) if a decarbonylation process could regulate the carbonylation modification, and (2) to which point the oxidant amplification loop will be forced to stop.

## 4. Na/K-ATPase Signaling and pNaKtide: A Specific Antagonist of c-Src Kinase that Breaks the Oxidant Amplification Loop

**Figure 1:**
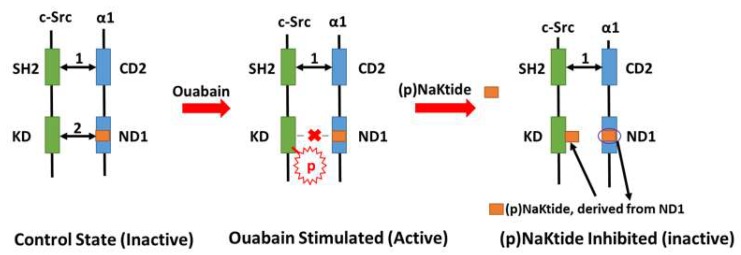
A schematic illustration of action of pNaKtide: Under control state, c-Src SH2 domain binds to α1 CD2 segment (indicated by arrow 1) and c-Src KD binds to α1 ND1 (indicated by arrow 2), which keeps c-Src inactive. Upon ouabain binding to the α1 subunit, the α1 subunit favors E-2P conformational status and c-Src KD released from α1 subunit that leads to phosphorylation of Tyr418 in c-Src KD. NaKtide and pNaKtide are derived from 20 aa (Ser415-Gln434) in α1 ND1, which can bind to the c-Src KD for the competitive binding of α1 ND1 and KD, thus preventing phosphorylation of Tyr418 in c-Src KD. In the illustration, ouabain is used as a representative of cardiotonic steroids. SH2, c-Src SH2 domain; KD, c-Src kinase domain; CD2, α1 subunit CD2 segment; ND1, α1 subunit ND1 segment.

One question regarding the abovementioned “Na/K-ATPase signaling-mediated oxidant amplification loop” is, could this amplification loop be controlled and targeted for possible therapeutic implication(s)? In the Na/K-ATPase/c-Src signaling complex model, it was demonstrated that the α1 ND1 domain binds to the c-Src tyrosine kinase domain and the α1 CD2 domain binds to the c-Src SH2 domain in the “resting” state [55]. Upon ouabain stimulation, c-Src is activated (phosphorylation of Tyr418) due to the disruption of the binding between the α1 ND1 domain and the c-Src tyrosine kinase domain. Based on this working model, mapping of these domains led to the identification of a peptide named NaKtide (derived from the Ser415-Gln434 of the pig α1 ND1 domain). In order to further explore this relationship, a cell-permeable version of NaKtide, named pNaKtide, was created. A 13-amino-acid TAT leader sequence makes pNaKtide positive and therefore cell permeable. pNaKtide targets the α1/Src receptor complex close to the plasma membrane inside the cell [41,56]. Both NaKtide and pNaKtide act as specific antagonists of c-Src phosphorylation (Figure 1), further demonstrating that the binding of the α1 and c-Src and the conformational change are critical in the activation of Na/K-ATPase signaling.

Oxidative stress plays an important role in many pathophysiological conditions. The role of pNaKtide in Na/K-ATPase signaling-mediated oxidant amplification loop has been investigated in different cell types and animal models. For example, systemic administration of pNaKtide significantly and effectively attenuates (1) 5/6th partial nephrectomy (PNx)-induced uremic cardiomyopathy phenotypes in C57BL/6 mice [57]; (2) high-fat-diet-induced adipogenesis, a model of obesity and metabolic syndrome [58]; (3) Western-diet-induced (containing high fat and high fructose) obesity, hepatic steatosis, and fibrosis in C57BL/6 mice, as well as steatohepatitis and aortic atherosclerosis in ApoE knockout mice [59]; (4) a Western-diet-accelerated aging process involving nuclear oxidative stress in C57BL/6 mice [60]; as well as (5) unilateral ureteral obstruction (UUO)-mediated interstitial fibrosis in C57BL/6J mice [61]. In these animal models, administration of pNaKtide specifically breaks Na/K-ATPase signaling-mediated oxidant amplification loop, demonstrated by pNaKtide-induced attenuation of c-Src activation, protein carbonylation, and other regulations. More molecular mechanistic studies are necessary for possible therapeutic usage.

## 5. Na/K-ATPase Signaling-Mediated Transporter Endocytosis and Renal Sodium Handling

Over the last decade, the role of Na/K-ATPase signaling in renal proximal tubular sodium handling and the role of oxidative modification of the Na/K-ATPase α1 subunit in Na/K-ATPase signaling were explored both in vitro and in vivo. The findings may explain some mechanism(s) related to the Na/K-ATPase signaling–ROS amplification loop and subsequent regulation of salt-sensitivity.

It is well documented that the renal proximal tubule mediates over 60% of the filtered Na^+^ reabsorption, mainly through apical Na^+^ entry via NHE3 and basolateral Na^+^ extrusion through the Na/K-ATPase. Coordinated and coupled regulation of NHE3 and the Na/K-ATPase is critical in maintaining intracellular Na^+^ homeostasis and extracellular fluid volume [62,63,64].

In LLC-PK1 cells such as dopamine [65,66,67,68], low concentrations of ouabain stimulate endocytosis of the α1/β1 subunits, NHE3 (Na^+^/H^+^ exchanger, isoform 3), and c-Src into early and/or late endosomes, leading to net decreases in abundance of Na/K-ATPase and NHE3 in cell surface, and thus decreases in transcellular ^22^Na^+^ transport [62,69,70,71,72,73,74,75]. This phenomenon is mainly through a clathrin-dependent endocytic pathway and requires caveolin-1 and activation of c-Src and PI3K. Furthermore, ouabain-induced endocytosis of Na/K-ATPase and NHE3 decreases in transcellular ^22^Na^+^ reabsorption and is dependent on the ouabain-stimulated signaling function of Na/K-ATPase without significantly affecting [Na^+^]_i_ [70,76,77]. Inhibition of c-Src and PI3K activity prevented ouabain-induced endocytosis of Na/K-ATPase and NHE3. Pretreatment of LLC-PK1 cells with membrane-permeable Ca^2+^ chelator BAPTA-AM attenuated ouabain-induced regulation of NHE3 [77], suggesting ouabain-induced Ca^2+^ signaling might be involved in regulation [14]. In male Sprague-Dawley rats fed a high-salt (4.0% NaCl) or normal-salt (0.4% NaCl) diet for 1 week, a high-salt diet redistributes the Na/K-ATPase α1 subunit from plasma membrane fraction to early/late endosomes, accompanied by a reduction of proximal tubular Na/K-ATPase ion-exchange activity and enzymatic activity but an increase in urinary excretion of marinobufagenin (MBG) and sodium. These effects were attenuated by administration of anti-MBG antibody prior to salt load [74]. Moreover, this observation was further confirmed in vivo. By using Dahl salt-sensitive and salt-resistant rats (Jr strains) as models, in vivo studies demonstrated that impairment of renal proximal tubular Na/K-ATPase signaling causes experimental Dahl salt sensitivity [78]. In Dahl salt-resistant but not salt-sensitive rats, a high-salt (2% NaCl, 1 week) diet activated proximal tubular Na/K-ATPase signaling and stimulated coordinated redistribution of the Na/K-ATPase and NHE3, leading to increases in total and fractional urinary sodium excretion as well as normal blood pressure. However, the mechanism(s) underlying the difference of Na/K-ATPase signaling function between Dahl salt-sensitive and salt-resistant rats, as well as the translation of Na/K-ATPase signaling to NHE3 regulation, are still unclear.

It is well established that both oxidative stress and high blood pressure are causes and consequences of each other. Based on the findings of the amplification loop of Na/K-ATPase signaling and ROS generation, we tested whether oxidative stress could activate the signaling function of Na/K-ATPase and induce the abovementioned endocytosis process and regulation of renal sodium handling. In our working model, increases in ROS generation, either by ouabain or by other stimuli such as glucose oxidase, are critical in activation of Na/K-ATPase signaling, which mediates transporter trafficking, transcellular Na^+^ transport, and urinary sodium excretion [45,46]. On the one hand, pretreatment with antioxidant NAC abrogates ouabain-stimulated Na/K-ATPase signaling and transcellular Na^+^ transport, suggesting that a certain level of basal ROS is required for initiation of Na/K-ATPase signaling. On the other hand, without the presence of ouabain, increases in ROS by extracellularly added glucose oxidase are able to activate Na/K-ATPase signaling, indicating that activation of Na/K-ATPase signaling does require its specific ligands and that general stimuli, such as oxidative modification alone, are able to activate Na/K-ATPase signaling [79].

However, the effect(s) and consequence(s) of ouabain- and ROS-induced endocytosis of Na/K-ATPase/c-Src/EGFR [70,71] are not clear. It has been shown that endocytosis of signaling molecules could be a way to terminate or propagate signaling and could further regulate endocytosis itself [80,81,82,83,84,85,86,87]. In this regard, it is possible that ouabain- and ROS-induced endocytosis could be an effective way to terminate the Na/K-ATPase signaling-mediated oxidant amplification loop by degradation of carbonylated Na/K-ATPase to maintain certain basal level of ROS and carbonylated protein [88].

## 6. Perspectives: The Working Models of Na/K-ATPase Signaling

**Figure 2:**
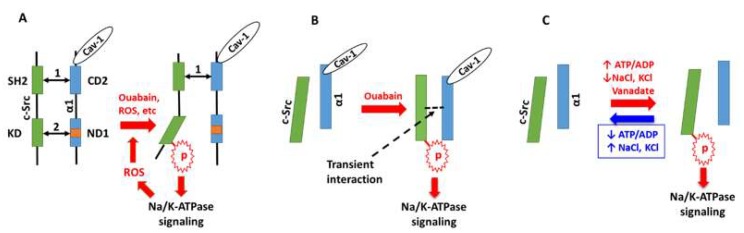
Schematic illustration of different working models. In the illustration, ouabain is used as a representative of cardiotonic steroids. (**A**) The model of Na/K-ATPase/c-Src (binding) receptor complex. Binding between c-Src SH2 domain and α1 CD2 segment as well as c-Src KD domain and α1 ND1 segment are indicated by arrow 1 and 2, respectively. (**B**) the model of c-Src activation by transiently binding to Na/K-ATPase/Cav-1 complex; (**C**) the model of c-Src activation regulated by the ATP/ADP ratio, ATPase inhibitor vanadate, and low concentrations of Na^+^ and K^+^. In this model, there is no binding between Na/K-ATPase and c-Src. The role of Cav-1 was not tested. Please refer to the references for details. Cav-1, caveolin-1; ROS, reactive oxygen species.

There are different proposed working models which explain the mechanisms underlying the activation of the Na/K-ATPase signaling function, including: (1) the direct interaction of the Na/K-ATPase α1 subunit with c-Src kinase which forms a functional Na/K-ATPase/c-Src signaling receptor complex, a model has been demonstrated both in vitro and in vivo [55,89,90,91]; (2) c-Src is activated primarily by an ATP-sparing effect (observed in a cell-free system) [92,93]; and (3) c-Src is activated by transient interaction with a Na/K-ATPase α1/caveolin-1 complex (also observed in a cell-free system) [94] (Figure 2). In these models, there is no doubt that c-Src activation is a proximal step in Na/K-ATPase signaling. It is not a surprise that different working models are proposed based on different experimental systems, and an ideal working model is developed based on new developments and new technologies. As mentioned above, ouabain (and other cardiotonic steroids), ROS, reactive nitrogen species (RNS), changes of ionic concentrations (bulky or local), and other stimuli can activate different signaling pathways to excute different functional regulations. Moreover, these different signaling pathways and functional regulations are also cell-dependent. A common charateristic in these working models is that they are, at least partially, dependent on the conformation change. Specifically, the E2-P conformational state of the Na/K-ATPase is favored and stabilized by Na/K-ATPase inhibitors (ouabain, vanadate, oligomycin), energy status (ATP/ADP ratio), and change in [Na^+^] and [K^+^]. While the E2-P conformational state of the Na/K-ATPase is favored, a “slower” dynamic E-2P ↔ E1-P conformational change (in the presence of inhibitors and/or energy status) might be an effective way to maintain and control the signaling strength and function. Nevertheless, these hypotheses need to be experimentally demonstrated.
